# Comparison Analysis of Machine Learning Techniques for Photovoltaic Prediction Using Weather Sensor Data

**DOI:** 10.3390/s20113129

**Published:** 2020-06-01

**Authors:** Berny Carrera, Kwanho Kim

**Affiliations:** Department of Industrial and Management Engineering, Incheon National University, Songdo 22012, Korea; berny@inu.ac.kr

**Keywords:** data mining, machine learning, weather sensors, forecasting solar power generation, deep neural networks

## Abstract

Over the past few years, solar power has significantly increased in popularity as a renewable energy. In the context of electricity generation, solar power offers clean and accessible energy, as it is not associated with global warming and pollution. The main challenge of solar power is its uncontrollable fluctuation since it is highly depending on other weather variables. Thus, forecasting energy generation is important for smart grid operators and solar electricity providers since they are required to ensure the power continuity in order to dispatch and properly prepare to store the energy. In this study, we propose an efficient comparison framework for forecasting the solar power that will be generated 36 h in advance from Yeongam solar power plant located in South Jeolla Province, South Korea. The results show a comparative analysis of the state-of-the-art techniques for solar power generation.

## 1. Introduction

### 1.1. Motivation

Nowadays, renewable energy is considered in different countries as source of electricity production. The principal characteristic of renewable energy is highly dependent on weather factors, making it difficult for obtaining a stable energy production. This characteristic leads to a production level that fluctuates with weather conditions [1]. Furthermore, power companies must guarantee a precise balance between the production and consumption of electricity. Therefore, they need to maintain a stability of services to their customers, forestalling unanticipated disturbances in the energy production. Furthermore, power companies must be prepared in advance to know the amount of energy that will possibly be produced in the next few hours. In general, a time horizon of 36 h ahead allows power companies to take optimal decisions and create a schedule for the generated energy. 

Although using various types of sensors easily captures weather data, the quantity and quality issues from those information create a PV forecasting challenge. For instance, solar power generation as renewable energy is highly dependent in the irradiance of the sun. To obtain an accurate prediction of solar power generation, the analysis of other weather factors is needed, such as whether it is a cloudy day or the humidity of the environment. Furthermore, a considerable amount of information related to the weather conditions are obtained every day from different meteorological stations. 

Additionally, it is important to understand what weather data (observable, forecasting or both) should be used and in what specific cases for better prediction results. Meteorological stations collect weather information from different weather sensors to forecast weather minutes, hours, or days ahead. The weather sensors gather information such as temperature, humidity, wind direction or solar irradiance useful to make predictions for renewable energy. Even though the solar power seems to be a bivariate forecast model (solar irradiance and temperature), it is subject to different factors such as cloudy weather, precipitation probability, humidity, solar position, etc. In addition, one major characteristic is that after being produced, it is difficult to store. For this reason, a proactive forecasting model is valuable for efficient management of energy where power companies can be prepared by enough resources to reduce electricity waste and improve and maintain the operations in the power system.

### 1.2. Literature Review

An analysis of the solar power forecasting is proposed in [2,3,4]. A classification can be made based on how the weather data are utilized as input. Three categories were observed, studies that only use weather forecast [5,6,7,8,9,10,11,12,13,14,15,16,17,18], those that use only weather observation [19,20,21,22,23,24,25], and those that use both forms of weather data [1,26,27,28,29,30]. In the first category, planning and projection is required before the actual generation of solar energy, but they are highly correlated with the errors that meteorological stations can make in the forecasting. The second category assumes that the changes in weather conditions may contain temporal interdependencies, and weather observations also tend to contain more variables than weather forecast. Lastly, the third category corrects errors in the predictions from weather forecast by weather observations.

In another classification, there exist many studies that estimate the relationship between solar power with weather data using machine learning approaches [2]. The methods used in other studies can be categorized into three categories: First are the single regression methods such as linear regression, Huber, ridge, lasso, elastic net, decision tree, K-nearest neighbors (k-NN) and support vector regression (SVR) [3,9,13,15,19,21,31]. For example, [19] uses an autoregressive (AR) model as a linear stationary model. In [9], the moving average (MA) method is used to analyze past solar power generation data by creating a series of averages of a different subsets, and double MA models are applied when a tendency in the values of the solar power generation is observed. In [31], k-NN, the autoregressive moving average (ARIMA), and artificial neural networks (ANN) are utilized. Second are the bagging ensemble models, bagging, random forest, and extra tress [25]. Finally, there are also the boosting ensemble models: adaboost, gradient boosting, catBoost, and XGBoost [12,24,32].

Some studies compare different machine learning algorithms such as linear regression, bagging, decision trees, random forests, support vector machines, and generalized additive models [1,3,21]. Regarding the studies that use similar data from South Korea, the study conducted in [1] uses the information from the same solar plant located in Yeongnam, making predictions day-ahead. Lee and Kim in [29] considered the solar power generation data from Gumi in South Korea. The approach in [16] implements a regression model for solar power generation given limited historical operational data. Their proposed model uses the information from the weather forecast and they considered cloud and temperature variables over thirty-two months of data. The drawback of this research is that they depend on the accuracy of the weather forecast provided by the Korea Meteorological Administration (KMA). Additionally, some variables are not taken in account such as the humidity, ground temperature, or solar elevation, which are important, highly correlated variables as presented in our research. In this study, the performance is compared with previously proposed methods for solar power forecasting.

### 1.3. Contribution and Paper Structure

In this study, we aim to conduct a comprehensive analysis of various up-to-date machine learning techniques to feature their pros and cons when they come to predict solar power generation problems. Specifically, we consider a particular prediction problem that is to predict solar power generation 36-h ahead using weather data, and we analyze the specific cases in which one must be used, such as the situations in which only one weather dataset is available in the region. The variables that are taken in account are the observed and the forecasted weather in order to gain an accurate prediction, and they can be beneficial for the grid operator and electricity supply companies. Additionally, the implementation and comparison of fifteen prediction methods (e.g., SVR, random forest, gradient boosting and XGboost) in three weather information settings (weather observation, weather forecasts and the combination of both) is taken into account. Furthermore, analysis and tuning of the parameters for each model are performed. The novelty of this paper is valuable to adopt and to replicate predictive methodologies based on the weather information for energy production power. Thus, the proposed methodology shows how solar power generation can be predicted using different types of weather variables. Furthermore, the methodology can be replicable regardless of geographic location with available weather data using only historical weather observations, forecasts, or both. In addition, this study presents how the maximum benefit can be obtained using a specific type of weather variables. Moreover, this study can be extended for the analysis of home solar power plants. 

This paper is organized as follows: Section 2 presents a brief overview of the utilized framework and the weather data; Section 3 describes the machine learning methods compared in this study; Section 4 presents the outcomes and the key results along with a comparison against the machine learning models; finally, Section 5 lists our discussion and future work.

## 2. Research Framework 

This section describes the framework and the weather datasets used to make a comparison for the prediction of solar power generation. Section 2.1 describes the framework with a brief description of the sources utilized in the study. Section 2.2 explains the preprocessing steps to prepare the data to be suitable for modelling. Section 2.3 presents the cross-validation step. Section 2.4 shows the comparison module utilizing ten-fold cross-validation. Finally, Section 2.5 introduce the subset selection. 

### 2.1. Framework and Data Collection

**Figure 1:**
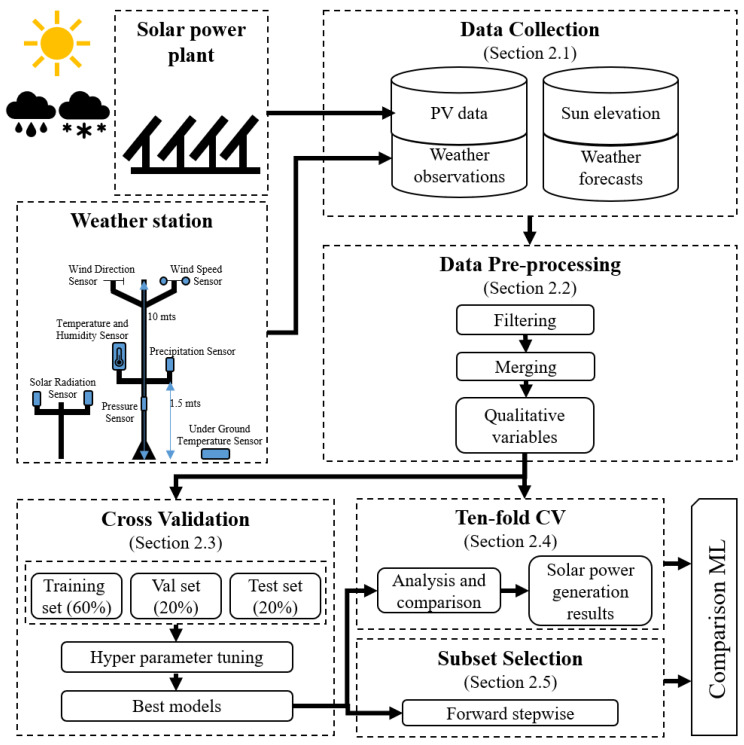
Proposed framework for the analysis and comparison of the machine learning techniques for photovoltaic prediction.

This subsection describes the data and the framework used to evaluate the machine learning methods tested in this study. Figure 1 presents a graphical representation of the evaluation framework. The framework consists in five steps: data collection, data preprocessing, cross-validation (CV), ten-fold cross-validation and subset selection. The data collection step gathers the information from four data sources, solar power generation data, solar elevation, observational and forecast weather data. 

Solar power generation data is provided by the Korean Open Data Portal (http://www.data.go.kr). Solar elevation data is given by the planetarium software Stellarium (http://www.stellarium.org) and observational and forecast weather data are offered by the Korea Meteorological Administration (KMA) (http://kma.go.kr). The solar power generation data used in this research is provided by the Korean government from Yeongam in South Korea. The solar power data is given in one-hour periods from 1 January 2013 to 31 December 2015.

**Figure 2:**
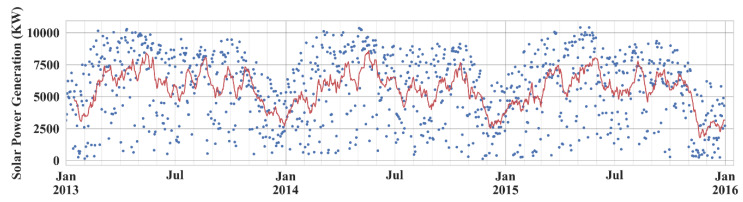
Solar power generation per day at Yeongam solar power plant from January 2013 to December 2015.

Figure 2 shows the total solar power generation per day in the three-year period, which implies the dynamic nature of solar power generation caused by weather conditions of each day. The graph shows the production levels fluctuate seasonally, where the greatest energy generation tends to be obtained in spring compared to the other seasons, and it tends to be the lowest in winter.

Along with the solar power generation data, we also collected three additional data that is to be used as the inputs of machine learning models. Firstly, the solar elevation data is taken from the geographical location of the power plant in Yeongam. The solar elevation represents the position of the sun from the power plant perspective in latitude and longitude coordinates. Next, the observational weather records provided by KMA are the measured actual weather. The observational weather data is taken from the closest meteorological station located in Mokpo, approximately 8.5 km from the PV plant. Finally, the forecast weather records announced by KMA are given in three-hour periods starting from 02:00 a.m. every day. In this study, the forecast weather data used are the predictions 36 h-ahead. 

The meteorological observation system utilized by KMA consists of a 10-m-high meteorological tower. At the top, wind direction and wind speed sensors are installed horizontally on the left and right sides. The humidity and temperature meter are installed at 1.5 m above the ground and a precipitation sensor is installed in the other side. A pressure sensor is installed around 50 to 60 cm above the ground.

### 2.2. Data Preprocessing

The preprocessing step prepares the gathered data to be suitable for the inputs of the models we considered. Firstly, in the filtering task, the instances with no sunlight information are excluded (00:00–05:00 and 20:00–24:00). Next, in the merging task, consecutive instances in three-hour periods are merged into a single instance, (1: 5 a.m.–8 a.m., 2: 8 a.m.–11 a.m., 3: 11 a.m.–2 p.m., 4: 2 p.m.–5 p.m., 5: 5 p.m.–8 p.m.), making a total of 5 records per day. In other words, data from solar power generation and weather observation are given in one-hour segments, and to match the weather forecast data, hourly data is aggregated every three hours. Additionally, the solar power generation is sliced in seven time periods so that the models can forecast each period one by one. Lastly, in the qualitative variables task, a conversion of all categorical variables to multiple binary variables is performed. Table 1 and Table 2 present the independent variables divided in three categories: solar elevation, weather forecast and weather observation. 

### 2.3. Cross-Validation

The cross-validation step is designed to obtain the best hyperparameter values of each model. In this step, different performance metrics are used to evaluate the models. First, a segmentation is performed on all data by dividing them sequentially into the training, validation, and test set (60%, 20%, 20%). The training set consists of data from 1 January 2013 to 19 October 2014. The validation set is composed of data from 20 October 2014 to 26 May 2015. Test set contains the information from 27 May 2015 to 31 December 2015. Second, to obtain the best hyper parameters, the grid-search method is utilized over training set and evaluated over validation set. In the test set, the first comparison of the generated models is made. Then the best performing models are sent as input for the last step. 

### 2.4. Ten-Fold CV

In the ten-fold cross-validation step, the complete data is taken as input and the generated 10 × 10 models from the previous step to analyze and evaluate each machine learning model power prediction for solar power generation. First, all the input data is randomly partitioned into ten equal segments. Second, from the ten segments, one is used as a validation set and the remaining nine segments are used as the training set. Third, the ten-fold cross-validation process is repeated ten times using each segment as a validation set. Last, the ten results are averaged to give a final result [33]. 

### 2.5. Subset Selection

Finally, in subset selection, we considered the forward stepwise method to select the best subsets from the independent variables. The forward stepwise propose a complexity of O(*N*^2^), smaller than O(2*^N^*) given by the best subset selection method. To choose the optimal model from the forward stepwise method, we analyzed four approaches: *C_p_*, Akaike’s information criterion (AIC), Bayesian information criterion (BIC), and adjusted *R*^2^ [34]. The criterion was performed with the training, validation, and test set from the cross-validation step, and these four approaches were then analyzed. Forward stepwise as methodology first starts modelling without any features. Following this, it tests the addition of each feature by calculating the performance metrics *C_p_*, AIC, BIC or adjusted *R*^2^, and then adds the feature that has the most statistically performance value. It subsequently repeats the process until the last feature, and finally selects the best performance subset from the complete dataset.

## 3. Machine Learning Methods

This section describes the methods tested in this study. For this approach, supervised learning algorithms for multiple independent variables are evaluated and categorized into three sections. Single regression models, bagging ensemble methods and boosting ensemble methods analyzed in this study are shown in Table 3.

### 3.1. Single Regression Methods

Linear regression is a simple yet useful approach in many various applications, and it is used as a base line in this study. By using the single regression model, the final PV output prediction for a particular time is calculated as follows:

y = *β*_0_ + *β*_1_*X*_1_ + … + *β_i_X_i_* + *ε*, (1)

where *X_j_* represents the *j*th dependent variable, and *β_j_* explains how much the variation in the outcome can be explained by the variation in the independent variable.

Moreover, the Huber, ridge, lasso and elastic net shrinking methods are involved, which use least squares to fit a linear model using a technique that regularized the coefficient estimates towards zero [34]. First, Huber regression is defined as a linear regression model that has robustness to outliers by minimizing the squared error and the absolute loss. Second, the ridge regression method focuses on solving the overfitting problem. Ridge is a regularized linear regression for analyzing multicollinearity and utilizes an *L*_2_ penalty to the square of the magnitude *β* as regularization. Next, least absolute shrinkage and selection operator (lasso) is one of the variants of the shrinkage methods and is an alternative to ridge regression. The principal problem of ridge algorithms is that they include all *X* predictors because *β* converges to zero, but this is not the case for lasso where *β* can be equal to zero. Thus, lasso selects variables from a subset of predictors *X*. Lasso regression utilizes the *L*_1_ penalty as a regularization method. Finally, the elastic net regression method arises from the limitation of the lasso regression methods where in a large subset of predictors *X* selects at most *n*, which is less than complete subset *i*. To overcome this limitation, elastic net utilizes the *L*_1_ and *L*_2_ penalty as regularization methods.

Other simple methods considered in this study are k-NN regression and SVR. k-nearest neighbors (k-NN) regression is a simple algorithm as it does not use a discriminative function but memorizes all available cases. This method chooses the number of k subsets and a distance metric to group cases into k subsets based on their similarity, and it finds the nearest neighbors based on the distance metric. In other words, k-NN regression predicts the average of solar power based on the similarity measure of the k subsets. On the other hand, SVR is a regression method that maintains all the characteristics from support vector machines. SVR tries to maximize the distance between the separating hyperplane and the support vectors within a threshold value. 

Decision tree methods are considered as one of the most popular algorithms in supervised learning in which their goal is to segment the predictors into several simple or smaller groups useful for interpretation [33]. As an example, for predicting the solar power generation 36 h-ahead, the decision tree starts to split all data by considering each *X* predictor where the mean of the subset is considered as the splitting value. One of the disadvantages of decision trees is that they are unstable with small variations in the predictors and can overfit the generated model. The variance of the model needs to be reduced, and this is achieved with other ensemble methods like bagging and boosting.

### 3.2. Bagging Ensemble Methods

The purpose of an ensemble of regressions is to find a more effective model by cooperating with a set of regression models by combining their results. Specifically, bagging ensemble methods try to decrease the model’s variance and train weak models in parallel. The most widely bagging method used is random forest [33]. Random forest constructs multiple decision trees as regression methods at training time and gains a mean prediction as an output. Another bagging method is extra trees, which like random forest, ensembles individual decision trees at training time and gains a mean prediction as an output. The principal difference is that each decision tree uses the full training subset and the splitting of the decision tree is randomized.

### 3.3. Boosting Ensemble Methods

Boosting ensemble algorithms try to decrease the model’s bias and they train different models sequentially in order to improve each previous model generated. In this study, state-of-the-art boosting methods will be used such as AdaBoost, CatBoost, gradient boosting and XGBoost. AdaBoost utilizes decision trees with a single split. 

Gradient boosting is different to AdaBoost in that it tries to fit the new decision tree model with the *y_i_*–*ŷ_i_* made by the previous decision tree model. CatBoost and XGboost are based on gradient boosting. CatBoost is a method that can support categorical variables as well as numerical variables. XGboost in comparison with gradient boosting uses a better regularized method to control overfitting. 

## 4. Experiment Results

This section presents the results of the machine learning methods described in the previous section. The results are grouped by the input weather data: observational, forecast and the combination of observational-forecast weather data. 

### 4.1. Performance Metrics

Section 4.1 presents the performance metrics used in this research. This study realizes a two-step analysis using k-fold cross-validation. The principal objective of cross-validation is understanding how well the model will make predictions over unseen data, indicating problems such as bias or overfitting [35].

To make a comparison of the algorithms based on the results obtained, it is important to estimate how a predictive model will perform in practice based on several performance metrics. The root mean square error (RMSE) is a metric that penalizes regression models for how far the *ŷ_i_* is from the *y_i_*, by squaring and averaging over *N*. The mean absolute error (MAE) measures the average distance between *ŷ_i_* and *y_i_*. The *R*^2^ measures the strength of the correlation between *ŷ_i_* and *y_i_*. We consider these three metrics because energy power prediction is a regression problem. RMSE penalizes the higher difference between prediction and actual solar power, and MAE directly takes the average of offsets. The mathematical equations of the performance metrics used in this study are as follows:

RMSE = (1/*N* × ∑ (*y_i_* − *ŷ_i_*)^2^)^1/2^, (2)

MAE = 1/*N* × *∑|y_i_* − *ŷ_i_*|, (3)

*R*^2^ = 1 − (∑ (*y_i_* − *ŷ_i_*)^2^ / ∑ (*y_i_* − *ӯ_i_*)*^2^*), (4)

where *y_i_* is the actual solar power value, *ŷ_i_* is the prediction value from the models, *ӯ_i_* is the mean of *y_i_*, and *N* is the sample size. 

To measure the subsets of the resulting models and to analyze the most important variables that improve the performance of the models, four approaches where compared: Mallows *C_p_* (*C_p_*), Akaike’s information criteria (AIC), Bayesian information criteria (BIC) and adjusted *R*^2^, represented as follows:

*C_p_* = 1/*N* (MSE × *N* + 2*dσ*^2^), (5)

AIC = 1/(*N* × *σ^2^*) (MSE × *N* + 2*dσ*^2^), (6)

BIC= 1/(*N* × *σ^2^*) (MSE × *N* + log(*N*) × *dσ*^2^), (7)

Adjusted R^2^ = 1 − (1 − R^2^) × (1 − *N*) / (*N* − *d* − 1), (8)

where MSE is the square of RMSE, *d* is number of features in our model and *σ*^2^ is the estimated standard error of the mean. AIC is a metric to compare other models, it tests the fitting of the data without overfitting and penalizes if is too complex. *C_p_* is a metric that makes a penalization when additional variables are added to the model and is a variant of AIC. BIC is a variant of AIC that has a stronger penalty for including additional variables. 

### 4.2. Cross-Validation

In this section, the data is separated in tree segments (training, validation, test). Furthermore, a grid search is performed to obtain the best models by obtaining the best hyper parameters values over the training set, and these models are compared over the test set.

The three years data used in the experiments is divided into three segments, training set (60%), validation set (20%) and test set (20%). To train the machine learning models, the training set was used, and as a resample procedure, the five-fold cross-validation was performed over the training time. Moreover, a grid search process was performed to find the best hyperparameter values for each model. The experiments were implemented using scikit-learn and statsmodels in Python 3.6, which offer implementations of those machine learning methods. The hyperparameter tuning processes are set to be identical to the ones for all data inputs, as shown in Table A1 in Appendix A.

Table 4 lists the tested hyperparameter candidates for each algorithm with each model’s best values. A total of eight single regression algorithms, three bagging ensemble algorithms and five boosting ensemble algorithms were analyzed. It should be noted that the hyperparameters present similar selection between the three datasets. Moreover, forecast data for single regression models and a decision tree model presents the best *R*^2^ in the test set with a maximum depth of = 3. XGBoost presents the best performance in the three datasets with a number of estimators = 80.

**Figure 3:**
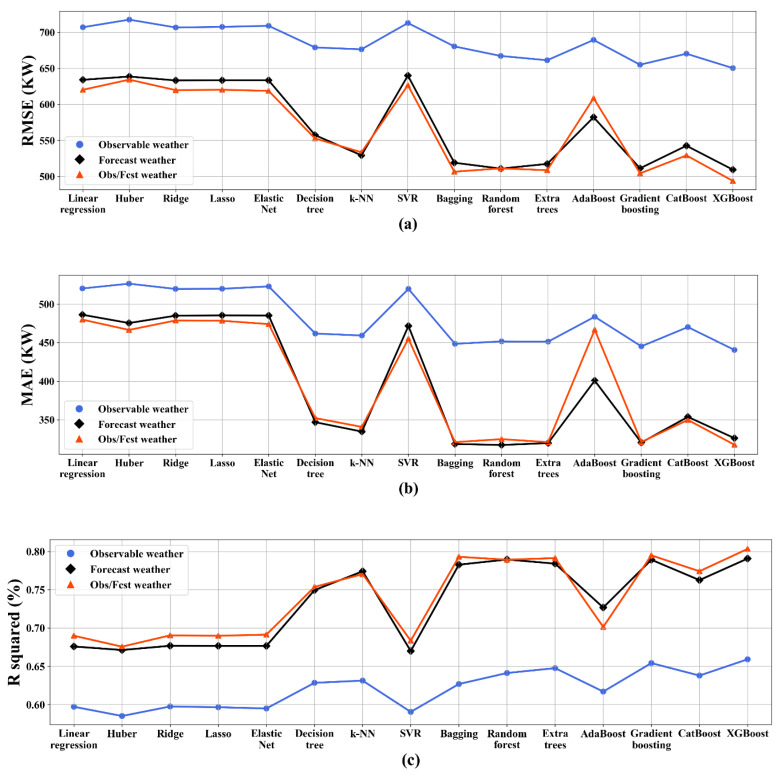
Comparative algorithm analysis using test set over weather data. (**a**) RMSE, (**b**) MAE, (**c**) *R*^2^.

Figure 3 presents the summary of the results of the comparative algorithm analysis over observational, forecast, and combined weather data. The lower part of Table A2 presents the performance of the fifteen models using observational and forecast weather. Regarding the results, the first group based on observational weather data, the single regression models, and the k-NN model showed the best performance in terms of RMSE, MAE and *R*^2^ for the test set (RMSE = 676.44, MAE = 459.42 and *R*^2^ = 63.1%). Among the ensemble models, gradient boosting and XGBoost yielded better performance than the single regression models. Between the ensembles, the XGBoost (RMSE = 650.36, MAE = 440.67, and *R*^2^ = 65.9%) performed better than gradient boosting. In the second group based on forecast weather data in single regression models, k-NN model showed the best performance in the three-performance metrics for the test set (RMSE = 529.37, MAE = 334.78, and *R*^2^ = 77.4%). Among the ensemble models, random forest obtained the best MAE for the test set (MAE = 317.40) and XGBoost showed the best performance in terms of RMSE and *R*^2^ for the test set (RMSE = 509.44 and *R*^2^ = 79.1). Finally, in the third group using observational-forecast weather data in single regression models, k-NN showed the best performance for the test set (RMSE = 533.59, MAE = 340.86, and *R*^2^ = 77.1%). Regarding the ensemble models, the XGBoost (RMSE = 493.85, MAE = 317.70, and *R*^2^ = 80.4%) performed better than the other models in terms of all performance measures, and it is also the only method that shows *R*^2^ value higher than 80%.

### 4.3. 10-Fold CV

In the current section, a ten-fold cross-validation was performed over all data using the best models obtained in the previous step. Additionally, a further analysis comparing the variance and the standard deviation of the model’s results were conducted over the three weather datasets.

The ten-fold cross-validation was performed over the dataset from 2013 to 2015 and the utilized machine learning models were the ones with the best hyperparameters obtained in the previous step. The goal of this step is to obtain the least biased machine learning model. Three analysis were implemented using weather observation, weather forecast and both. The analysis was performed over ten runs for each machine learning method, making comparative statistics for each regression model and obtaining the mean and the standard deviation for each performance metric. Furthermore, the results were separated in single regression models and ensemble models based on their interpretability. 

**Figure 4:**
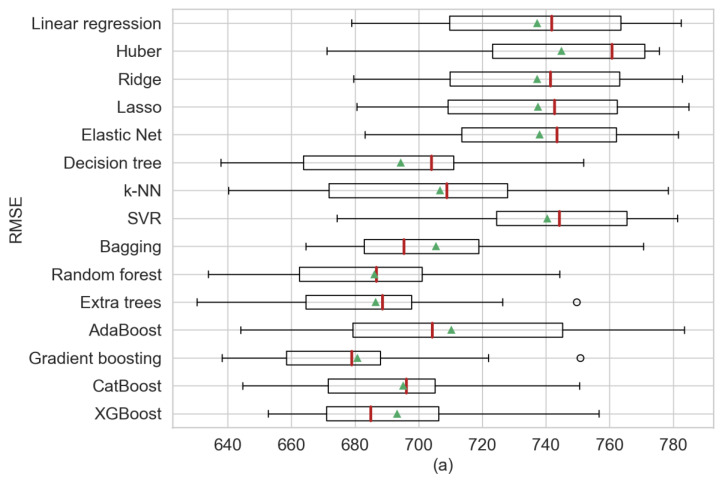

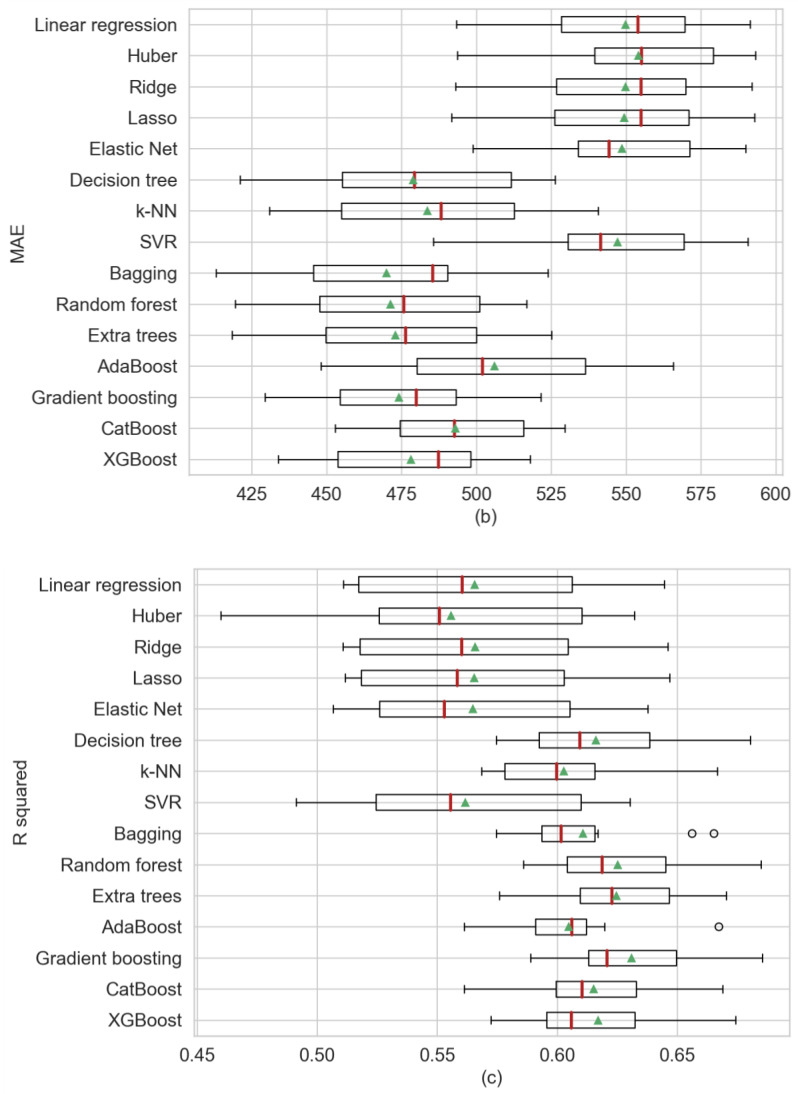
Comparative algorithm analysis using 10-fold cross-validation over observational weather data analyzing (**a**) RMSE, (**b**) MAE, (**c**) *R*^2^ as scoring metric.

**Figure 5:**
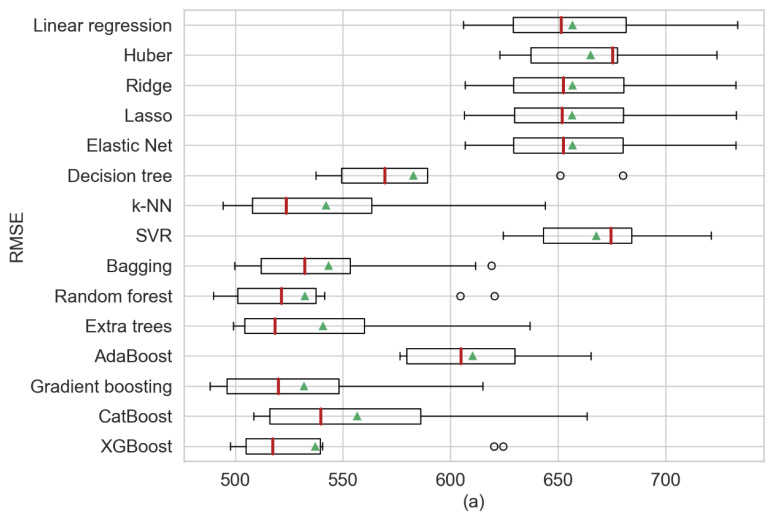

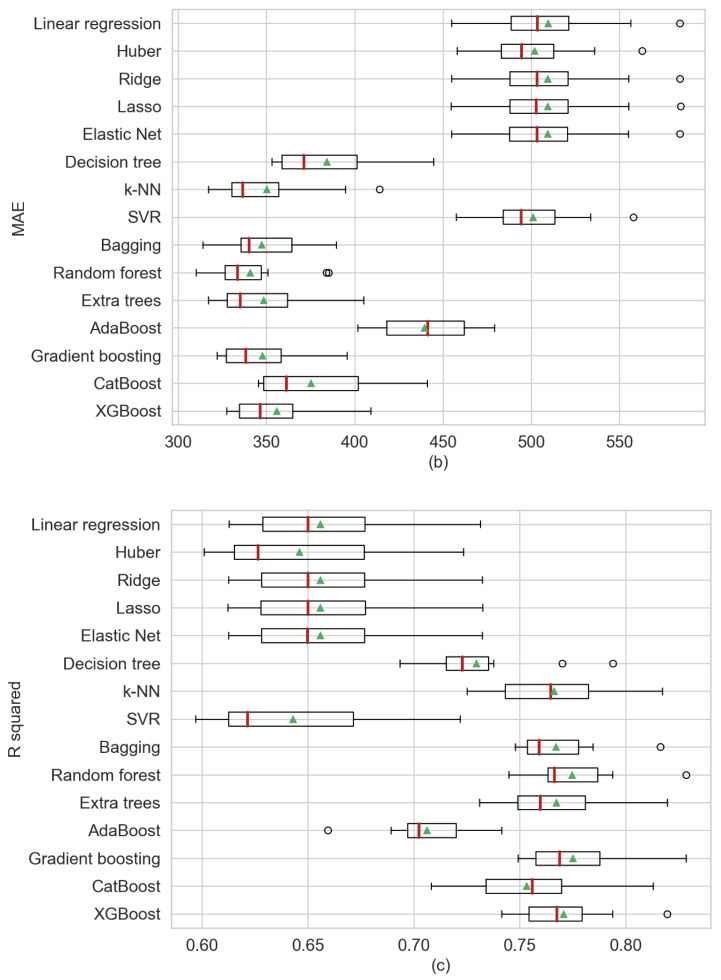
Comparative algorithm analysis using 10-fold cross-validation over forecast weather data analyzing (**a**) RMSE, (**b**) MAE, (**c**) *R*^2^ as scoring metric.

**Figure 6:**
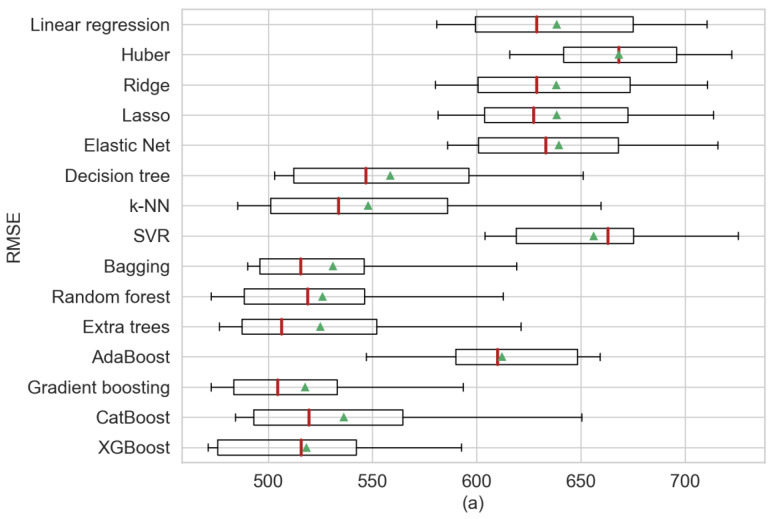

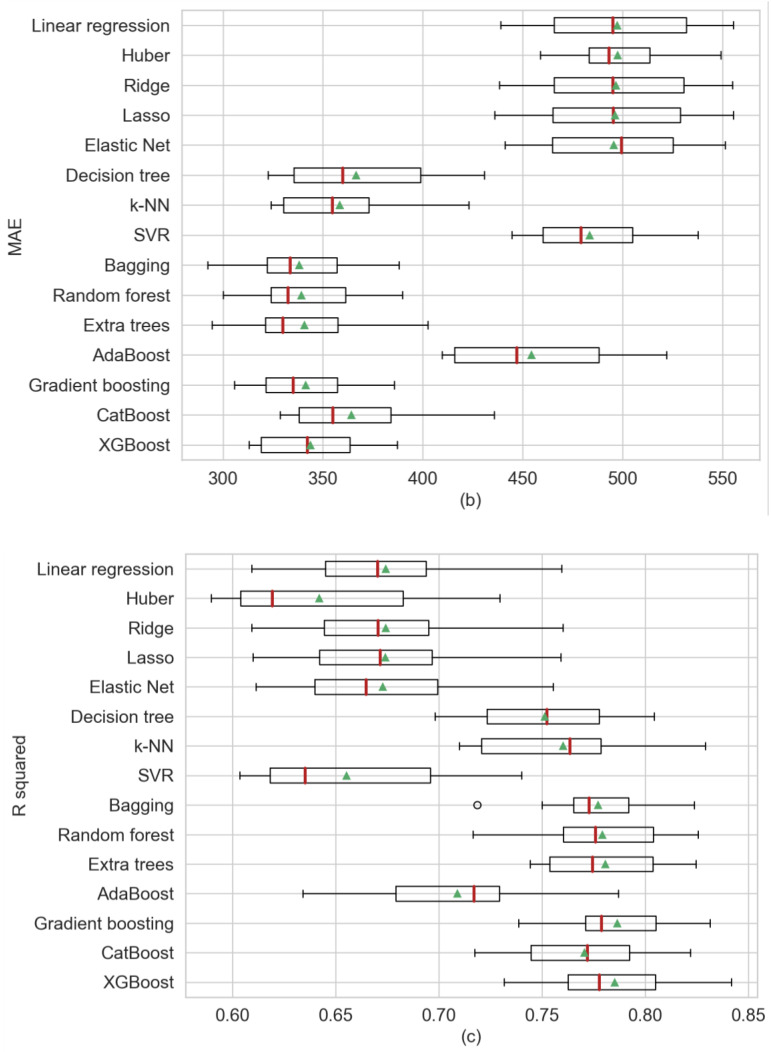
Comparative algorithm analysis using 10-fold cross-validation over observable and forecast weather data analyzing (**a**) RMSE, (**b**) MAE, (**c**) *R*^2^ as scoring metric.

The first analysis presented in Table 5 provides a comparison of the statistical information of the model’s regression using three perspectives: RSME, MAE and *R*^2^ from which the mean and the standard deviation (STD) were obtained. For Figure 4, Figure 5 and Figure 6, the red line represents the medians and the green triangle the means. In our results, for the single regression model’s decision trees obtained the best performance, outperforming k-NN in terms of mean. Additionally, elastic net presented the least biased model. Furthermore, gradient boosting obtained the best scores or RMSE and MAE in comparison with XGBoost that presented better results in the previous step. XGBoost achieved the least biased model based on the standard deviation. It can be observed that in the single regression model, the decision tree showed the best performance in regard to RMSE, MAE and *R*^2^ in terms of their means (RMSE = 694.24, MAE = 478.77 and *R*^2^ = 61.6%). Among the ensemble models, gradient boosting yielded the best RMSE and *R*^2^ in terms of their means (RMSE = 680.65 and *R*^2^ = 63.1%) and bagging regression shows the best MAE (MAE = 470.1). Figure 4 shows three boxplots with the results of the machine learning models separated by each performance metric. Additionally, in Figure 4a, the median for gradient boosting and how the predictions shown a negative skewed distribution can be observed.

The second analysis showed in Table 6 presents the comparative statistics using weather forecast data. Similar to the results obtained from cross-validation, in single regression models, k-NN presents the best statistics in the three performances metrics based on the means (RMSE = 542.09, MAE = 350.20 and *R*^2^ = 76.6%) and SVR obtained the smaller standard deviation in RMSE and MAE. For the ensembles models, on one hand similar to the weather observation data, gradient boosting presents the best results for RMSE and *R*^2^ in terms of mean (RMSE = 531.85 and *R*^2^ = 77.5%), while on the other hand, random forest present the best results in MAE performance in terms of mean (MAE = 340.77). Figure 5 illustrates the results gained after applying ten-fold cross-validation. For this experiment, gradient boosting shows a better performance over k-NN, but it can have observed that the mean of random forest varies much less than gradient boosting. Random forest has a more consistent mean relative to the weather forecast.

Finally, the third analysis presents the results of using observation and forecast weather data, as shown in Table 7. For the single regression models, k-NN gained the best scores—similar to when using forecast weather data. The k-NN model yielded better performances in RMSE, MAE and *R*^2^ based on their mean (RMSE = 547.79, MAE = 358.28 and *R*^2^ = 76.0%). Among the ensemble models, gradient boosting and bagging yielded better performance than the single regression models. Between the ensembles, the gradient boosting (RMSE = 517.56, R2 = 78.6%) performed better than bagging (MAE = 338.03). Figure 6 presents the statistical results and it can be observed that gradient boosting has the best mean in RMSE and *R*^2^ compared with other models. Furthermore, it can be observed in Figure 6b that bagging and gradient boosting have a similar prediction distribution. Moreover, in Figure 6a, the difference is more noticeable.

### 4.4. Subset Selection

**Figure 7:**
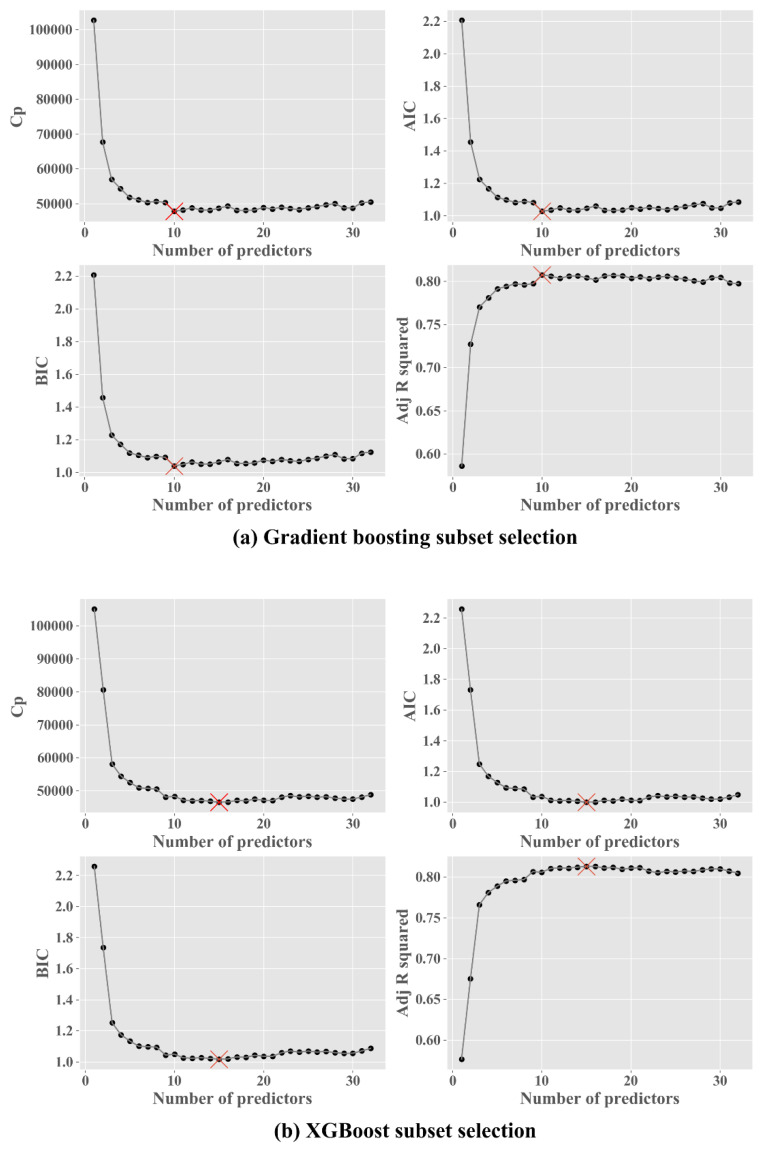
Forward stepwise subset selection using *C_p_*, AIC, BIC and adjusted *R*^2^.

**Figure A1:**
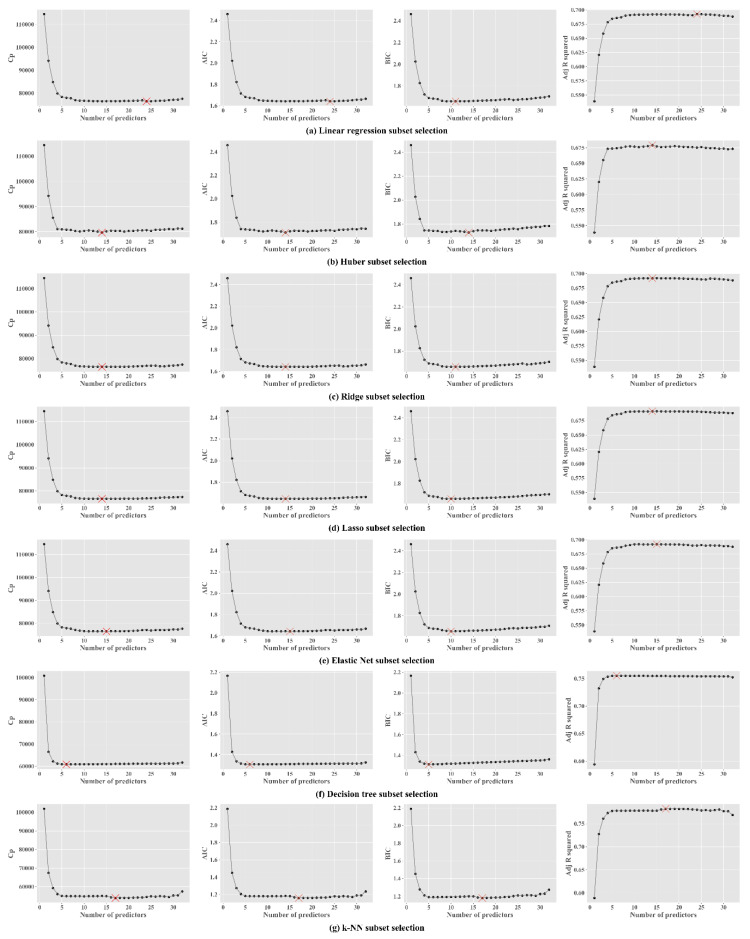

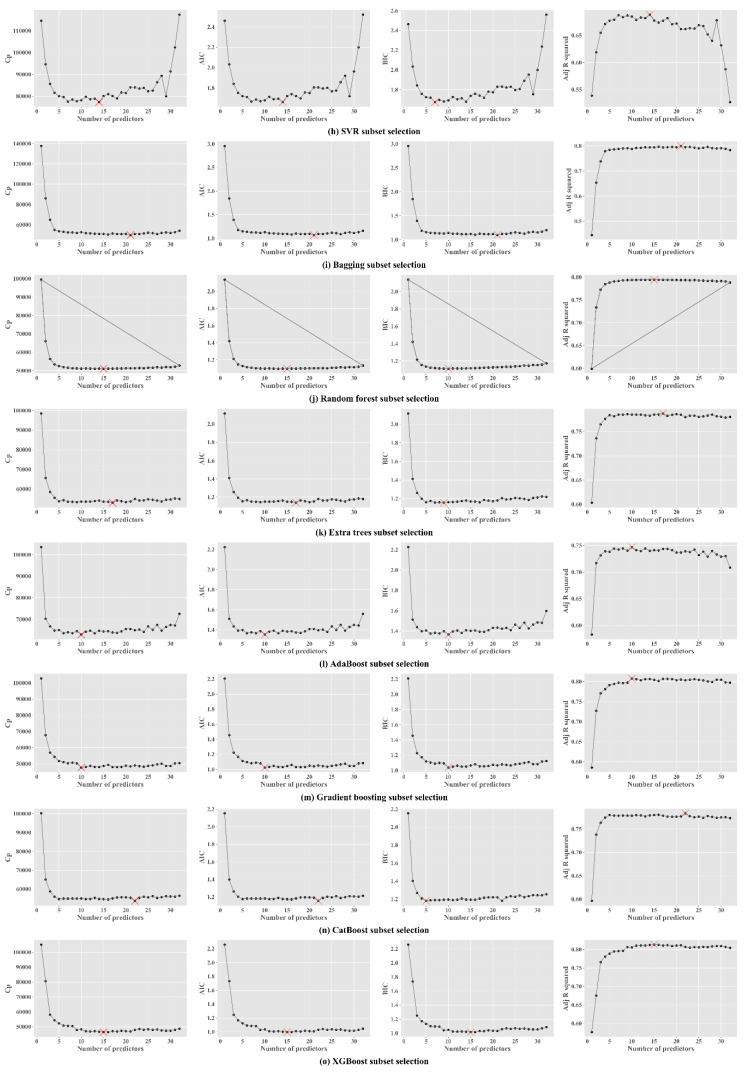
Forward stepwise subset selection using *C_p_*, AIC, BIC and adjusted *R*^2^.

This subsection presents the results from the forward stepwise. The goal of this step is finding the optimal combination of features of the compared models. As explained previously, RMSE, MAE and *R*^2^ are sensitive to the addition of more features in the model. Thus, we use the *C_p_*, AIC, BIC and adjusted *R*^2^, which penalizes the inclusion of features to the model. The results of all the models are shown in Figure A1. Figure 7 present the best two algorithms from cross-validation and the ten-fold CV step. In our experiments, gradient boosting and XGBoost present the best values in adjusted *R*^2^. Gradient boosting obtained the minimum values of *C_p_* = 47831.4, AIC = 1.027 and BIC = 1.040 and the maximum value of adjusted *R*^2^ = 80.8% at ten number of predictors. Table 8 present the ten features used to obtain the optimal gradient boosting model. XGboost gained the minimum values of *C_p_* = 46528.2, AIC = 0.999 and BIC = 1.018 and the maximum value of adjusted *R*^2^ = 81.3% at 15 features. Table 9 present the 13 variables and the 15 features obtained to gain the optimal model from XGBoost.

**Figure 8:**
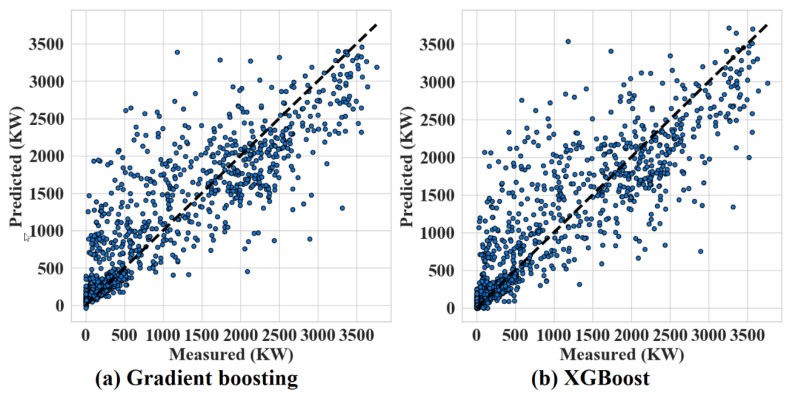
Performances of gradient boosting and XGBoost from 27 May 2015 to 31 December 2015.

Figure 8 draws the comparison of gradient boosting and XGBoost in the test set (27 May 2015 to 31 December 2015). In Figure 8, some similarities of the dispersion of the scatter plots can be observed. Furthermore, differences can be observed, for example, XGBoost can effectively predict higher peaks of energy (>3500 KW) and it does not predict negative values (<0 KW) in comparison with gradient boosting predictions.

## 5. Discussion and Conclusions

In this study, we proposed a comparative of different machine learning techniques for the prediction of solar power generation 36 h ahead. The input and output are in a time frame of 3 h (1: 5 a.m.–8 a.m., 2: 8 a.m.–11 a.m., 3: 11 a.m.–2 p.m., 4: 2 p.m.–5 p.m., 5: 5 p.m.–8 p.m.), making a total of five records per day, and they are the same in all the methods considered in this study. The proposed study uses an input data time frame of 3 h similar with [1,6,11,16,30] and the region of this study is Asia [1,5,9,15,16,30]. One characteristic considered for predicting solar power energy 36 h-ahead is the weather observation, because it has a negative correlation with solar power generation. For example, solar power generation energy predictions made at 5:00 a.m. in the morning would be for 5:00 p.m. the next day. Furthermore, the number of weather variables that can be obtained to predict energy must be considered. Therefore, we performed experiments using weather forecast, weather observation and the combination of both. 

Additionally, a framework with five steps is proposed: data collection, data preprocessing, cross-validation, ten-fold CV and selection subset. First, we suggest a comparative using cross-validation and ten-fold CV because by training the model only with a training set, we cannot be sure of the desired variance and accuracy that will be obtained. In cross-validation, XGBoost performed better than the other algorithms, and in ten-fold CV, gradient boosting gained the best performance and generally in a less biased model. Therefore, these two algorithms must be studied in regard to the implementations of prediction of photovoltaic energy, and they should be compared with future proposals. Moreover, as general interpretations, some differences and similarities in the performances can be observed: for example in Figure 4, Figure 5 and Figure 6, some single models present a similar performance, and this may be due to the fact that their primal formula is based on linear regression methods such as Huber, ridge, lasso, elastic net and SVR. Additionally, the assumption of the data can be another characteristic that can differentiate the power of prediction, for example, the closeness in time can affect the variance in these models. Furthermore, gradient boosting and XGBoost show the optimal models in the subset selection step. In the subset selection step, the metrics *C_p_*. AIC, BIC and adjusted *R*^2^ are metrics for optimal model selection, and these metrics are used directly with the training set. In our study, we considered analyzing these four metrics in the subset selection to evaluate if there exists any difference, but as it could be seen in our experiments with gradient boosting and XGBoost, the four converged on the same subset features. 

In regard to the limitations of this study, the results are based on the weather-relevant data, and previous solar power data is not taken into account because we want to emphasize the power prediction of the weather data. In addition, for this study, only machine learning regression algorithms are analyzed—no other time series algorithms were examined. Furthermore, based on the comparative analysis presented in this study, future studies can adopt analysis of the other time series algorithms such as ARIMA, or deep learning techniques such as artificial neural networks or long short-term memory. Finally, by using a single photovoltaic plant, the methodology can easily be replicated for the analysis of other photovoltaic plants in other countries, that is, the methodology can be replicated in different places where only one type of weather data can be obtained. Future studies can support solar power forecasting for different time frames such as 48 or 72 h-ahead, or for different input data time frames such as 30 min or 1 h.

## Figures and Tables

**Table 1:** Solar elevation and weather forecast variables.

Category	Variable Name	Classification	Description (Unit)
Solar elevation (1)	Elevation	Continuous	Degrees (0°–76°)
Weather forecast (# variables: 7) (# features: 16)	Humidity	Continuous	(%)
	PrecipitationProb	Continuous	(%)
	PrecipitationCat	Categorical	0: none, 1: rain, 2: sleet, 3: snow
	SkyType	Categorical	0: clear sky, 1: slightly cloudy, 2: partly cloudy, 3: overcast
	Temperature	Continuous	Celsius (°C)
	WindDirection	Categorical	N: 315°–45°, E: 45°–135°, W: 225°–315°, S: 135°–225°
	WindSpeed	Continuous	(m/s)

**Table 2:** Weather observation variables.

Category	Variable Name	Classification	Description (Unit)
Weather observation (# variables: 16) (# features: 19)	AirTemperature	Continuous	(°C)
	AtmosPressure	Continuous	(hPa)
	DewPointTemperature	Continuous	(°C)
	GroundTemperature	Continuous	(°C)
	Humidity	Continuous	(%)
	Precipitation	Continuous	(mm)
	SeaLevelPressure	Continuous	(hPa)
	SolarRadiation	Continuous	(MJ/m^2^)
	SunlightTime	Continuous	(hr)
	VaporPressure	Continuous	(hPa)
	WindDirection	Categorical	N: 315°–45°, E: 45°–135°, W: 225°–315°, S: 135°–225°
	WindSpeed	Continuous	(m/s)
	5cmDownTemperature	Continuous	Temperature below the ground surface (°C)
	10cmDownTemperature	Continuous	
	20cmDownTemperature	Continuous	
	30cmDownTemperature	Continuous	

**Table 3:** Machine learning methods.

Single Regression	Ensemble (Bagging)	Ensemble (Boosting)
Linear regression	Bagging	AdaBoost
Huber	Random forest	Gradient boosting
Ridge	Extra trees	CatBoost
Lasso		XGBoost
Elastic Net		
Decision tree		
k-NN		
SVR		

**Table 4:** Best values for the evaluated hyperparameter tuning from grid search.

Prediction Models		Observation Weather	Forecast Weather	Forecast and Observation Weather
Single regression models	Linear regression	N/A	N/A	N/A
	Huber	α = {**0.1**}	α = {**0.01**}	α = {**10**}
	Ridge	α = {**0.8**}	α = {**2**}	α = {**1.0**}
	Lasso	α = {**0.1**}	α = {**1**}	α = {**1**}
	Elastic net	α = {**0.0001**}, max_iterations = {**10**}, *l*_1_ = {**0.9**}	α = {**0.001**}, max_iterations = {**10**}, *l*_1_ = {**0.7**}	α = {**0.0001**}, max_iterations = {**10**}, *l*_1_ = {**0.9**}
	Decision tree	max_depth = {**3**}	max_depth= {**6**}	max_depth = {**5**}
	k-NN	*k* = {**15**}	*k* = {**15**}	*k* = {**15**}
	SVR	*C* = {**10**}, γ = {**0.001**}	*C* = {**10**}, γ = {**0.001**}	*C* = {**10**}, γ = {**0.001**}
Ensemble models (bagging)	Bagging	num_estimators = {**80**}	num_estimators = {**80**}	num_estimators = {**80**}
	Random forest	max_depth = {**7**}, num_estimators = {**80**}	max_depth = {**7**}, num_estimators = {**80**}	max_depth = {**7**}, num_estimators = {**80**}
	Extra trees	max_depth = {**7**}, num_estimators = {**80**}	max_depth = {**9**}, num_estimators = {**80**}	max_depth = {**7**}, num_estimators = {**20**},
	AdaBoost	N/A	N/A	N/A
Ensemble models (boosting)	Gradient boosting	max_depth = {**5**}, num_estimators = {**80**}	max_depth = {**5**}, num_estimators = {**80**}	max_depth = {**7**} num_estimators = {**80**}
	CatBoost	iterations = {**50**}, learning_rate = {**0.1**}, depth = {**3**}	iterations = {**50**}, learning_rate = {**0.1**}, depth = {**3**}	iterations = {**50**}, learning_rate = {**0.1**}, depth = {**3**}
	XGBoost	num_estimators = {**80**}	num_estimators = {**80**}	num_estimators = {**80**}

**Table 5:** Comparative statistics of prediction models based on observation weather information using 10-fold cross-validation.

Prediction Models		RMSE		MAE		*R* ^2^	
		Mean	STD	Mean	STD	Mean	STD
	Linear regression	737.22	32.76	549.73	30.44	0.5656	0.0467
Single regression models	Huber	744.82	32.95	554.09	29.9	0.5557	0.0561
	Ridge	737.15	32.65	549.69	30.95	0.5656	0.0466
	Lasso	737.49	32.75	549.37	31.48	0.5653	0.0465
	Elastic net	737.9	29.79	548.56	28.3	0.5648	0.0449
	Decision tree	694.24	35.83	478.77	34.68	0.616	0.0312
	k-NN	706.65	42.12	483.64	36.71	0.6027	0.0299
	SVR	740.4	32.1	547.15	29.98	0.5615	0.0497
Ensemble models (bagging)	Bagging	705.42	34.71	470.01	33.18	0.6106	0.0275
	Random forest	685.97	33.71	471.31	33.8	0.6251	0.029
	Extra trees	686.43	32.87	472.94	34.51	0.6245	0.0295
	AdaBoost	710.19	43.01	506	38.73	0.6047	0.0266
Ensemble models (boosting)	Gradient boosting	680.65	33	474.09	28.3	0.6309	0.028
	CatBoost	695.07	33.49	492.89	26.7	0.6151	0.0294
	XGBoost	693.13	32.75	478.05	26.16	0.617	0.0318

The best values are in underlined boldface.

**Table 6:** Comparative statistics of prediction models based on forecast weather information using 10-fold cross-validation.

Prediction Models		RMSE		MAE		*R* ^2^	
		Mean	STD	Mean	STD	Mean	STD
	Linear regression	656.62	36.88	509.51	35.74	0.6558	0.0357
Single regression models	Huber	665.09	30.53	501.79	28.61	0.6460	0.0414
	Ridge	656.65	36.61	509.17	35.60	0.6557	0.0362
	Lasso	656.54	36.73	509.18	35.68	0.6558	0.0364
	Elastic net	656.62	36.57	509.07	35.58	0.6557	0.0362
	Decision tree	582.60	45.43	384.15	32.12	0.7295	0.0299
	k-NN	542.09	47.76	350.20	29.78	0.7661	0.0276
	SVR	667.79	29.18	500.79	27.48	0.6429	0.0430
Ensemble models (bagging)	Bagging	543.32	39.60	347.18	25.03	0.7671	0.0203
	Random forest	532.20	43.19	340.77	24.11	0.7746	0.0230
	Extra trees	540.77	46.86	348.29	28.70	0.7673	0.0262
	AdaBoost	610.32	32.46	439.47	26.32	0.7061	0.0232
Ensemble models (boosting)	Gradient boosting	531.85	42.73	347.75	24.67	0.7749	0.0227
	CatBoost	556.66	49.60	375.19	32.40	0.7532	0.0301
	XGBoost	537.06	44.69	355.67	28.67	0.7707	0.0219

The best values are in underlined boldface.

**Table 7:** Comparative statistics of prediction models based on observation and forecast weather information using 10-fold cross-validation.

Prediction Models		RMSE		MAE		*R* ^2^	
		Mean	STD	Mean	STD	Mean	STD
Single regression models	Linear regression	638.18	43.81	497.20	38.50	0.6741	0.0440
	Huber	668.09	34.30	497.38	25.83	0.6419	0.0494
	Ridge	638.06	43.62	496.58	38.22	0.6742	0.0440
	Lasso	638.36	43.61	495.98	38.02	0.6740	0.0437
	Elastic net	639.37	42.45	495.47	35.45	0.6727	0.0445
	Decision tree	558.35	49.92	366.54	36.39	0.7512	0.0354
	k-NN	547.79	56.24	358.28	31.88	0.7601	0.0395
	SVR	656.00	38.92	483.36	29.68	0.6551	0.0472
Ensemble models (bagging)	Bagging	530.90	42.84	338.03	27.46	0.7770	0.0295
	Random forest	525.88	46.17	339.20	29.82	0.7791	0.0324
	Extra trees	524.92	48.44	340.73	31.94	0.7805	0.0287
	AdaBoost	612.09	38.51	454.29	40.33	0.7088	0.0416
Ensemble models (boosting)	**Gradient boosting**	517.56	42.09	341.22	24.99	0.7864	0.0267
	CatBoost	536.23	52.59	364.20	32.35	0.7704	0.0353
	XGBoost	518.30	43.45	343.65	25.59	0.7850	0.0324

The best values are in underlined boldface.

**Table 8:** Gradient boosting weather variables.

Category	Variable Name	Classification	Description (unit)
Solar elevation (1)	Elevation	Continuous	Degrees (0°–76°)
Weather forecast (Features: 5)	Humidity	Continuous	(%)
	PrecipitationProb	Continuous	(%)
	PrecipitationCat	Categorical	0: none
	Temperature	Continuous	Celsius (°C)
	WindSpeed	Continuous	(m/s)
Weather observation (Features: 4)	AtmosPressure	Continuous	(hPa)
	SolarRadiation	Continuous	(MJ/m^2^)
	WindDirection	Categorical	W: 225°–315°
	5cmDownTemperature	Continuous	Temperature below the ground surface (°C)

**Table 9:** XGboost weather variables.

Category	Variable Name	Classification	Description (unit)
Solar elevation (1)	Elevation	Continuous	Degrees (0°–76°)
Weather forecast (Features: 8)	Humidity	Continuous	(%)
	PrecipitationProb	Continuous	(%)
	PrecipitationCat	Categorical	1: rain
	Temperature	Continuous	Celsius (°C)
	WindDirection	Categorical	E: 45°–135°, W: 225°–315°, S: 135°–225°
	WindSpeed	Continuous	(m/s)
Weather observation (Features: 6)	Humidity	Continuous	(%)
	SolarRadiation	Continuous	(MJ/m^2^)
	WindDirection	Categorical	S: 135°–225°
	GroundTemperature	Continuous	Celsius (°C)
	5cmDownTemperature	Continuous	Temperature below the ground surface (°C)
	20cmDownTemperature	Continuous	

## Appendix A

**Table A1:** Hyperparameter tuning using grid search for the machine learning algorithms.

Input Data	Prediction Models		Evaluated Hyperparameters
Observational data	Single regression models	Linear regression	N/A
		Huber	α = {0.0001, 0.001, 0.01, **0.1**, 1, 10, 100}
		Ridge	α = {0.01, 0.02, 0.05, 0.1, 0.2, 0.3, 0.5, **0.8**, 1.0, 2, 4, 10, 25}
		Lasso	α = {0.0001, 0.001, 0.01, **0.1**, 1, 10, 100}
		Elastic net	α = {**0.0001**, 0.001, 0.01, 0.1, 1, 10, 100}, max_iterations = {1, 5, **10**}, *L*_1_= {0.0, 0.1, 0.2, 0.3, 0.4, 0.5, 0.6, 0.7, 0.8, **0.9**}
		Decision tree	max_depth = {1, 2, **3**, 4, 5, 6, 7, 8, 9, 10}
		k-NN	*k* = {5, 10, **15**, 20, 40, 80}
		SVR	*C* = {0.001, 0.01, 0.1, 1, **10**}, γ = {**0.001**, 0.01, 0.1, 1}
	Ensemble models (bagging)	Bagging	num_estimators = {5, 10, 15, 20, 40, **80**}
		Random forest	max_depth = {2, 5, **7**, 9}, num_estimators = {5, 10, 15, 20, 40, **80**}
		Extra trees	max_depth = {2, 5, **7**, 9}, num_estimators = {5, 10, 15, 20, 40, **80**}
		AdaBoost	N/A
	Ensemble models (boosting)	Gradient boosting	max_depth = {2, **5**, 7, 9}, num_estimators = {5, 10, 15, 20, 40, **80**}
		CatBoost	iterations = {**50**, 100, 1000}, learning_rate = {0.0001, 0.001, 0.01, **0.1**}, depth = {2, **3**, 4}
		XGBoost	num_estimators = {5, 10, 15, 20, 40, **80**}
Forecast data		Linear regression	N/A
		Huber	α = {0.0001, 0.001, **0.01**, 0.1, 1, 10, 100}
	Single regression models	Ridge	α = {0.01, 0.02, 0.05, 0.1, 0.2, 0.3, 0.5, 0.8, 1.0, **2**, 4, 10, 25}
		Lasso	α = {0.0001, 0.001, 0.01, 0.1, **1**, 10, 100}
		Elastic net	α = {0.0001, **0.001**, 0.01, 0.1, 1, 10, 100}, max_iterations = {1, 5, **10**}, *L*_1_ = {0.0, 0.1, 0.2, 0.3, 0.4, 0.5, 0.6, **0.7**, 0.8, 0.9}
		Decision tree	max_depth= {1, 2, 3, 4, 5, **6**, 7, 8, 9, 10}
		k-NN	*k* = {5, 10, **15**, 20, 40, 80}
		SVR	*C* = {0.001, 0.01, 0.1, 1, **10**}, γ = {**0.001**, 0.01, 0.1, 1}
	Ensemble models (bagging)	Bagging	num_estimators = {5, 10, 15, 20, 40, **80**}
		Random forest	max_depth = {2, 5, **7**, 9}, num_estimators = {5, 10, 15, 20, 40, **80**}
		Extra trees	max_depth = {2, 5, 7, **9**}, num_estimators = {5, 10, 15, 20, 40, **80**}
		AdaBoost	N/A
	Ensemble models (boosting)	Gradient boosting	max_depth = {2, **5**, 7, 9}, num_estimators = {5, 10, 15, 20, 40, **80**}
		CatBoost	iterations = {**50**, 100, 1000}, learning_rate = {0.0001, 0.001, 0.01, **0.1**}, depth = {2, **3**, 4}
		XGBoost	num_estimators = {5, 10, 15, 20, 40, **80**}
Forecast and observational data	Single regression models	Linear regression	N/A
		Huber	α = {0.0001, 0.001, 0.01, 0.1, 1, **10**, 100}
		Ridge	α = {0.01, 0.02, 0.05, 0.1, 0.2, 0.3, 0.5, 0.8, **1.0**, 2, 4, 10, 25}
		Lasso	α = {0.0001, 0.001, 0.01, 0.1, **1**, 10, 100}
		Elastic net	α = {**0.0001**, 0.001, 0.01, 0.1, 1, 10, 100}, max_iterations = {1, 5, **10**}, *L*_1_ = {0.0, 0.1, 0.2, 0.3, 0.4, 0.5, 0.6, 0.7, 0.8, **0.9**}
		Decision tree	max_depth = {1, 2, 3, 4, **5**, 6, 7, 8, 9, 10}
		k-NN	*k* = {5, 10, **15**, 20, 40, 80}
		SVR	*C* = {0.001, 0.01, 0.1, 1, **10**}, γ = {**0.001**, 0.01, 0.1, 1}
	Ensemble models (bagging)	Bagging	num_estimators = {5, 10, 15, 20, 40, **80**}
		Random forest	max_depth = {2, 5, **7**, 9}, num_estimators = {5, 10, 15, 20, 40, **80**}
		Extra trees	max_depth = {2, 5, **7**, 9}, num_estimators = {5, 10, 15, **20**, 40, 80},
		AdaBoost	N/A
	Ensemble models (boosting)	Gradient boosting	max_depth = {2, 5, **7**, 9} num_estimators = {5, 10, 15, 20, 40, **80**}
		CatBoost	iterations = {**50**, 100, 1000}, learning_rate = {0.0001, 0.001, 0.01, **0.1**}, depth = {2, **3**, 4}
		XGBoost	num_estimators = {5, 10, 15, 20, 40, **80**}

The best values are in underlined boldface.

**Table A2:** Performances of solar power prediction models.

Input Data	Prediction Models			Validation Set			Test Set		
				RMSE	MAE	*R* ^2^	RMSE	MAE	*R* ^2^
Observational data	Single regression models	Linear regression	Hyperparameters	735.04	547.74	59.8%	707.08	520.44	59.7%
		Huber	α = 0.1	746.26	554.02	58.6%	717.63	526.67	58.5%
		Ridge	α = 0.8	735.06	547.32	59.8%	706.76	519.81	59.8%
		Lasso	α = 0.1	734.49	545.77	59.9%	707.61	520.03	59.7%
		Elastic net	α = 0.0001, *L*_1_ = 0.9, max_iterations = 10	734.66	541.45	59.9%	709.04	523.10	59.5%
		Decision tree	max_depth = 3	685.75	478.41	65.1%	679.07	461.94	62.9%
		k-NN	*k* = 15	683.80	475.89	65.3%	676.44	459.42	63.1%
		SVR	*C* = 10, γ = 0.001	741.87	546.58	59.1%	712.95	519.84	59.1%
	Ensemble models (bagging)	Bagging	num_estimators = 80	685.02	466.09	65.1%	680.51	448.63	62.7%
		Random forest	max_depth = 7, num_estimators = 80	669.35	466.51	66.7%	667.26	451.78	64.1%
		Extra trees	max_depth = 7, num_estimators = 80	684.45	478.97	65.2%	661.29	451.48	64.8%
		AdaBoost	N/A	707.59	507.44	62.8%	689.47	483.75	61.7%
	Ensemble models (boosting)	Gradient boosting	max_depth = 5, num_estimators = 80	673.19	473.28	66.3%	655.07	445.37	65.4%
		CatBoost	depth = 3, iterations = 50, learning_rate = 0.1	690.14	497.52	64.6%	670.36	470.36	63.8%
		**XGBoost**	num_estimators = 80	681.32	474.58	65.5%	650.36	440.67	65.9%
Forecast data	Single regression models	Linear regression	N/A	657.30	509.97	67.9%	634.18	486.35	67.6%
		Huber	α = 0.01	671.02	505.89	66.5%	638.68	475.59	67.1%
		Ridge	α = 2.0	658.26	510.17	67.8%	633.29	485.14	67.7%
		Lasso	α = 1.0	657.79	509.97	67.8%	633.44	485.52	67.7%
		Elastic net	α = 0.001, *L*_1_ = 0.7, max_iterations = 10	658.03	510.02	67.8%	633.46	485.34	67.7%
		Decision tree	max_depth = 6	576.69	376.62	75.3%	557.41	346.96	75.0%
		k-NN	*k* = 15	537.32	358.39	78.5%	529.37	334.78	77.4%
		SVR	*C* = 10, γ = 0.001	678.00	507.18	65.8%	640.00	471.79	67.0%
		Bagging	num_estimators = 80	527.20	337.95	79.3%	519.11	318.74	78.3%
		Random forest	max_depth = 7, num_estimators = 80	518.98	335.68	80.0%	510.96	317.40	79.0%
		Extra trees	max_depth = 9, num_estimators = 80	537.02	351.18	78.6%	517.46	319.89	78.4%
		AdaBoost	N/A	595.58	430.29	73.6%	582.24	400.90	72.7%
		Gradient boosting	max_depth = 5, num_estimators = 80	514.93	344.69	80.3%	511.35	320.56	78.9%
		CatBoost	depth = 3, iterations = 50, learning_rate = 0.1	543.21	372.91	78.1%	542.67	353.85	76.3%
		**XGBoost**	num_estimators = 80	525.21	349.87	79.5%	509.44	326.25	79.1%
Forecast and observational data	Single regression models	Linear regression	N/A	637.29	496.72	69.8%	620.31	480.03	69.0%
		Huber	α = 10	670.20	500.31	66.6%	634.42	466.65	67.6%
		Ridge	α = 1.0	637.95	496.44	69.8%	619.79	478.94	69.1%
		Lasso	α = 1.0	638.54	495.73	69.7%	620.35	478.54	69.0%
		Elastic net	α = 0.0001, *L*_1_ = 0.9, max_iterations = 10	639.79	494.39	69.6%	618.82	474.22	69.2%
		Decision tree	max_depth = 5	531.96	355.22	79.0%	552.80	352.34	75.4%
		k-NN	*k* = 15	548.95	368.00	77.6%	533.59	340.86	77.1%
		SVR	*C* = 10, γ = 0.001	660.39	487.39	67.6%	626.68	455.21	68.4%
	Ensemble models (bagging)	Bagging	num_estimators = 80	505.33	323.03	81.0%	506.64	321.09	79.3%
		Random forest	max_depth = 7, num_estimators = 80	503.64	330.35	81.2%	511.29	325.00	78.9%
		Extra trees	max_depth = 7, num_estimators = 20	512.54	340.11	80.5%	508.68	321.05	79.2%
		AdaBoost	N/A	622.54	487.11	71.2%	608.63	466.95	70.2%
	Ensemble models (boosting)	Gradient boosting	max_depth = 7, num_estimators = 80	501.84	336.85	81.3%	504.33	321.25	79.5%
		CatBoost	depth = 3, iterations = 50, learning_rate = 0.1	521.38	365.95	79.8%	529.27	349.92	77.4%
		**XGBoost**	num_estimators = 80	518.16	340.40	80.0%	493.85	317.70	80.4%

The best values are in underlined boldface.
